# PHYSICAL CAPACITY AS MARKER FOR RATE OF AGING IN MID LIFE

**DOI:** 10.1093/geroni/igad104.0913

**Published:** 2023-12-21

**Authors:** Roy Tzemah-Shahar, Maayan Agmon

**Affiliations:** University of Haifa, Ramat-Gan, HaZafon, Israel; University of Haifa, Haifa, HaZafon, Israel

## Abstract

Biological age captures the heterogeneity of aging by providing an estimation for rate of aging. As an alternative to the widely investigated laboratory biomarkers of aging, behavioral functional markers of physical capacity may offer a feasible alternative for identifying individuals at risk for negative aging trajectory. We aimed to examine the associations of different functional tests both together and as a composite score of physical capacity with rate of aging. We conducted a cross-sectional study, with midlife adults (age ~45) reporting being able to perform leisure physical exercise. Biological age was estimated using the Klemera-Doubal method and a set of physiological biomarkers; rate of aging, ∆Age, was defined as the difference between age and estimated biological age. Physical capacity was measured using a testing battery of 15 functional tests linked with six physical capacity domains. 116 participants completed the testing battery. For women, better results in terms of strength, flexibility, cardiorespiratory fitness, and balance tests were negatively correlated with ∆Age (r=0.38-0.29, p<0.05); for men, beyond these tests, agility was also negatively correlated with ∆Age (r=0.27-0.59, p<0.05). A sex-standardized composite score of physical capacity was negatively associated with ∆Age after controlling for chronological age, smoking, and education (r=-0.437, p=0.007; r=-0.491, p<0.001 for women and men respectively). The suggested physical capacity battery offers a functional assessment for ∆Age. Higher physical capacity metrics correlate with smaller ∆Age, corresponding with younger biological age. Measuring physical capacity may help to assess aging trajectory and offer a suitable behavioral intervention goal.

